# 3-Nitrooxypropanol substantially decreased enteric methane emissions of dairy cows fed true protein- or urea-containing diets

**DOI:** 10.1016/j.heliyon.2022.e09738

**Published:** 2022-06-16

**Authors:** Florencia Garcia, Camila Muñoz, Jorge Martínez-Ferrer, Natalie L. Urrutia, Emilio D. Martínez, Marcelo Saldivia, Irmgard Immig, Maik Kindermann, Nicola Walker, Emilio M. Ungerfeld

**Affiliations:** a Universidad Nacional de Córdoba, Facultad de Ciencias Agropecuarias, Departamento de Producción Animal, Ing Agr. Félix Aldo Marrone 746, Córdoba Capital, Córdoba, 5001, Argentina; b Instituto de Investigaciones Agropecuarias, Centro Regional de Investigación Remehue, Ruta 5 km 8 norte, Osorno, Los Lagos, 5290000, Chile; c Instituto Nacional de Tecnología Agropecuaria, Estación Experimental Agropecuaria Manfredi, Ruta Nacional nº9 km 636, Manfredi, Córdoba, 5988, Argentina; d Facultad de Ciencias Veterinarias, Universidad Austral de Chile, Campus Isla Teja S/N, Valdivia, 5090000, Chile; e DSM Nutritional Products, Animal Nutrition and Health, Wurmisweg 576, Kaiseraugst, 4303, Switzerland; f Instituto de Investigaciones Agropecuarias, Centro Regional de Investigación Carillanca, Camino Cajón - Vilcún km 10, Temuco, La Araucanía, 4880000, Chile

**Keywords:** Methane, Methanogenesis inhibition, Rumen, Ruminants, Nitrogen, Microbial protein

## Abstract

Methane is a potent but short-lived greenhouse gas targeted for short-term amelioration of climate change, with enteric methane emitted by ruminants being the most important anthropogenic source of methane. Ruminant production also releases nitrogen to the environment, resulting in groundwater pollution and emissions of greenhouse gas nitrous oxide. We hypothesized that inhibiting rumen methanogenesis in dairy cows with chemical inhibitor 3-nitrooxypropanol (3-NOP) would redirect metabolic hydrogen towards synthesis of microbial amino acids. Our objective was to investigate the effects of 3-NOP on methane emissions, rumen fermentation and nitrogen metabolism of dairy cows fed true protein or urea as nitrogen sources. Eight ruminally-cannulated cows were fed a plant protein or a urea-containing diet during a Control experimental period followed by a methanogenesis inhibition period with 3-NOP supplementation. All diets were unintentionally deficient in nitrogen, and diets supplemented with 3-NOP had higher fiber than diets fed in the Control period. Higher dietary fiber content in the 3-NOP period would be expected to cause higher methane emissions; however, methane emissions adjusted by dry matter and digested organic matter intake were 54% lower with 3-NOP supplementation. Also, despite of the more fibrous diet, 3-NOP shifted rumen fermentation from acetate to propionate. The post-feeding rumen ammonium peak was substantially lower in the 3-NOP period, although that did not translate into greater rumen microbial protein production nor lesser nitrogen excretion in urine. Presumably, because all diets resulted in low rumen ammonium, and intake of digestible organic matter was lower in the 3-NOP period compared to the Control period, the synthesis of microbial amino acids was limited by nitrogen and energy, precluding the evaluation of our hypothesis. Supplementation with 3-NOP was highly effective at decreasing methane emissions with a lower quality diet, both with true protein and urea as nitrogen sources.

## 1 Introduction

Methane (CH_4_)^2^ is a potent greenhouse gas 28 times stronger than carbon dioxide at trapping heat within a 100-year timeframe. Because CH_4_´s life-time in the atmosphere is considerably shorter than carbon dioxide's, mitigation of anthropogenic CH_4_ emissions is strategic for short-term amelioration of global warming (Saunois et al., 2016; Beauchemin et al., 2020). Enteric CH_4_ from rumen fermentation is the most important source of anthropogenic emissions of CH_4_ (E.P.A., 2012; Hristov et al., 2013). Methane emissions from ruminants are also an energy loss for ruminants, ranging between 2 and 12% of ingested gross energy (GE) (Johnson and Johnson, 1995). Thus, because of both environmental and animal production reasons, there is much interest in decreasing CH_4_ emissions from ruminants.

A second environmental issue associated to ruminant production is the release of excess nitrogen (N) to the environment. Rapid digestion of dietary protein and deamination of amino acids (AA) in the rumen, and intraruminal N recycling, can surpass the capacity of rumen microbes to incorporate ammonium (NH_4_^+^) into the synthesis of amino acids (AA), and excess NH_4_^+^ is absorbed through the rumen wall. Although part of the absorbed NH_4_^+^ is recycled back to the rumen as urea, most excess N is eliminated as urea in urine (Firkins, 1996; Wallace et al., 1997; Bach et al., 2005). In the soil, urea is rapidly hydrolyzed to NH_4_^+^, contaminating groundwater and producing nitrous oxide, a very potent greenhouse gas with a global warming potential 298 times greater than carbon dioxide in a 100-year scale (Eckard et al., 2010; Hristov et al., 2013). Elimination of excess N to the environment is particularly relevant in temperate regions such as southern Chile, where forages can have a high content of rumen degradable protein (Valderrama and Anrique, 2011).

Maximizing the incorporation of NH_4_^+^ into the synthesis of microbial AA in the rumen decreases the absorption of NH_4_^+^ through the rumen wall while increasing the supply of AA available for absorption at the small intestine (Wallace et al., 1997; Hartinger et al., 2018). At non-limiting NH_4_^+^ concentration, incorporation of NH_4_^+^ into carbon chains by the mixed rumen microbiota is predominantly catalyzed by low-affinity NAD(P)H-dependent dehydrogenases. Oxidative deamination, as the reverse reaction, necessitates the oxidized co-factors, NAD^+^ or NADP^+^, to act as electron acceptors (Wallace et al., 1997; Pengpeng and Tan, 2013). Inhibiting methanogenesis in vitro increased the NADH/NAD^+^ ratio and decreased deamination (Hino and Russell, 1985). It has been proposed that the incomplete recovery of metabolic hydrogen observed when rumen methanogenesis is inhibited is partly explained by the incorporation of NH_4_^+^ into carbon chains to synthesize microbial AA (Ungerfeld et al., 2007; Ungerfeld, 2015). More recent findings have confirmed that inhibiting methanogenesis stimulated the incorporation of NH_4_^+^ into microbial AA biosynthesis in rumen cultures grown on starch as the energy and carbon source, but not when grown on cellulose (Ungerfeld et al., 2019, 2020).

Urea is hydrolyzed in the rumen to NH_4_^+^ and can be supplemented to ruminant diets as a cheap source of non-protein N (Schwab and Broderick, 2017; Hailemariam et al., 2021). When rumen microbes are instead supplemented true protein as N source, they decrease the synthesis of AA from NH_4_^+^ and increase the proportion of microbial protein synthesized from direct incorporation of preformed AA (Atasoglu et al., 1999). We hypothesized that inhibiting methanogenesis would increase the synthesis of microbial AA from NH_4_^+^ with a diet containing urea as non-protein N but not with a plant protein-supplemented diet. 3-Nitrooxypropanol (3-NOP) is a small molecule consistently proven to be effective at inhibiting rumen methanogenesis (Dijkstra et al., 2018) in long term experiments (Hristov et al., 2015), with an established mechanism of action (Duin et al., 2016). The objective of this study was to investigate the effects of supplementing 3-NOP to dairy cows on CH_4_ production, rumen fermentation, and N metabolism with a urea-containing diet in comparison to a plant protein-based diet.

## 2 Methods

The experiment was conducted at Instituto de Investigaciones Agropecuarias (INIA), Centro Regional de Investigación Remehue (40°31′S; 73°03′W; 65 m above sea level) in Osorno, Chile, in October–November 2018. Cows were cared for in accordance to the requirements of the Chilean Law 20380 of animal protection, in accordance with the European Union Directive 2010/63/EU for animal experiments, and with the approval of the INIA ethics committee for animal care and use (Approval 02/2016 from June 2016). All animals remained healthy throughout the study.

### 2.1 Animals and experimental design

Eight ruminally-cannulated Holstein Friesian cows with an initial body mass of 456 ± 50.2 kg (mean ± SD) and 64.0 ± 6.1 d (mean ± SD) in milk were used in the study. Two different N sources (Plant protein or Urea) were evaluated under functional rumen methanogenesis conditions, and with rumen methanogenesis inhibited by 3-NOP supplementation.

Each cow remained with the same N source for the entire experiment, which lasted 32 d.

The study had two experimental periods. In the first period (Control period), all 8 cows received the 3-NOP carrier, composed by SiO_2_ and propylene glycol, as a placebo without 3-NOP (Hristov et al., 2015). In the second period (methanogenesis inhibition, or 3-NOP period), all eight cows received 3-NOP with its carrier at 100 mg/kg of total feed DM (Table 1). This design allowed animals to act as their own controls and to rule out carry over effects of 3-NOP (Martinez-Fernandez et al., 2018).

**Table 1:** Ingredient composition of diets formulated for dairy cows fed a plant protein only (Plant protein) or a urea-containing (Urea) diet, supplemented a placebo (Control period) or the methanogenesis inhibitor 3-nitrooxypropanol (3-NOP period).

N source	Plant protein		Urea	
Period	Control	3-NOP	Control	3-NOP
Ingredients, g/kg DM				
Grass silage^1^	474	474	478	478
Flaked corn	235	235	322	322
Rapeseed meal	274	274	171	171
Urea	0.00	0.00	11.6	11.6
Minerals and vitamins premix^2^	16.0	16.0	16.1	16.1
Placebo (3-NOP carrier, SiO_2_ and propylene glycol)	0.90	0.90	0.90	0.90
3-NOP	-	0.10	-	0.10

1 Silage was produced from a natural grassland in which predominated *Holcus lanatus*, *Lolium multiflorum*, *Poa* spp. and *Anthoxanthum odoratum*.

2 Nutrialmix Acid Buf, Santiago, Chile. Contains per kg: calcium 200 g, phosphorus 41 g, magnesium 58 g, sulfur 13 g, copper 1022 mg, manganese 1160 mg, zinc 2580 mg, iodine 95 mg, cobalt 21 mg, selenium 24 mg, vitamin A 400,000 IU, vitamin D3 80,000 IU, biotin 70 mg.

A pre-experimental period of 18 d for adaptation to the diets preceded the Control period. Both experimental periods included a 10-d subperiod of adaptation to the placebo or 3-NOP, followed by a 6-d measurements and sampling subperiod. The relatively short adaptation subperiod to methanogenesis inhibition by 3-NOP intended to decrease the difference in days in milk between the measurement and sampling subperiods of the Control and the 3-NOP periods to reduce confounding the effect of methanogenesis inhibition with differences in days in milk. Adaptation periods of 8 (Mitsumori et al., 2012) or 10 d (Lopes et al., 2016; Martinez-Fernandez et al., 2016) to small molecule inhibitors of methanogenesis have been successfully used in previous studies.

Cows were divided into two blocks of four cows each based on body mass, milk production and days in milk, and two cows of each block were randomly assigned to the Plant protein or the Urea diets. The diets were based on grass silage bales (about 500 kg DM), flaked corn, a mineral and vitamin premix, and rapeseed meal (Plant protein diet) or rapeseed meal and urea (Urea diet; Table 1). Silage was produced from a native grassland chiefly composed of *Holcus lanatus, Lolium multiflorum, Poa* spp, and *Anthoxanthum odoratum*. Diets were formulated to meet requirements for maintenance and milk production of cows by using The Spartan Ration Evaluator/Balancer for Dairy Cattle (2010), with a target content of net energy for lactation of 6.67 MJ/kg dry matter (DM), and a target content of 16% DM crude protein (CP), based on the proximate composition of composite samples of silage bales, flaked corn and rapeseed meal obtained previously to the beginning of the study.

### 2.2 Feeding and management

During the adaptation subperiods, each diet was fed to the corresponding four cows in one pen. During the measurement and sampling subperiod, cows were kept in individual tie stalls fitted with rubber mattresses and fed individually. Cows had continuous access to water throughout the experiment and were milked twice daily (5:30 and 16:00 h).

The concentrate fraction of each diet (the mixture of flaked corn, rapeseed meal, urea if applicable, minerals and vitamins premix, and the placebo or the formulated 3-NOP additive; from this point on, the “concentrate mixture”) was weekly premixed in a cement type mixer. The as-fed proportions of concentrate mixture and silage were calculated based on the DM content of the silage bales analyzed prior to the experiment, and the concentrate mixture and the grass silage were then manually mixed at each individual cow's feeder. The diets were offered to cows once daily in the morning, allowing for 10% feed refusals (as-fed basis) based on their intake from the previous day to ensure ad libitum feed intake.

### 2.3 Feeds, feces and urine sampling

All silage bales were sampled and analyzed for DM and CP before the experiment. In the measurement and sampling subperiods, silage offered and feed refusals of each individual cow were weighed and sampled every day. Daily samples of the concentrate mixture were composited per diet and per period on an equal mass basis. All samples of silage, concentrate mixture and refusals were kept frozen until analyzed.

On days 4–6 of the measurement and sampling subperiod, total collection of feces and urine was conducted as by Muñoz et al. (2019). Feces were collected in stainless steel trays (100 × 120 × 20 cm) lined with plastic placed behind each cow. Urine was collected in 25-L plastic containers via a flexible hose and funnel which was attached using Velcro to patches glued around the cow's vulva and rump. To minimize losses of N as ammonia, urine was acidified during collection with sulfuric acid (35% v/v) to maintain pH < 3.0 (Stevens et al., 2009). Total daily fecal and urinary outputs were weighed. Samples of 5% of total daily excretion (feces by mass and urine by volume) were obtained after thorough mixing and composited per cow per period. Samples of feces and urine were kept at -20 °C until chemical analyses.

### 2.4 Milk production and composition

Individual milk yields were recorded at each milking. Composite daily milk samples, obtained by mixing proportional volumes of the morning and afternoon milking, were collected on days 4–6 of the measurement and sampling subperiods, mixed with bronopol, and stored at 4 °C until analysis.

### 2.5 Methane measurements

Individual measurements of CH_4_ emissions were conducted by using the sulfur hexafluoride (SF_6_) tracer gas technique (Muñoz et al., 2019). Cows were dosed orally with calibrated permeation tubes (supplied by National Institute of Water & Atmospheric Research, Wellington, New Zealand) previously incubated at 39 °C for 7.5 wk and weighed twice weekly to calculate their rates of SF_6_ release (5.74 ± 0.081, 5.49–6.05 mg/d; mean ± SD, range). Boluses were paired by release rate and one bolus of each pair was randomly assigned to one cow per block and diet and placed into the rumen 1 wk before the beginning of the first measurement and sampling subperiod.

On the first day of each measurement and sampling subperiod, cows were fitted with a head collar with a sampling line placed above the animal's nostrils, which had a filter and a calibrated (∼0.30 mL/min) flow-restriction capillary tube connected to an air-evacuated V-shaped PVC canister (2.5 L) suspended above the metabolism stalls. Canisters had initial and final gas pressures of 1,104 ± 342 and 37,227 ± 2823 (mean ± SD) Pa, respectively. Canisters collected subsamples of exhaled and eructed gases for 24 h and were changed daily. Background concentration of CH_4_ and SF_6_ in the barn were determined using four sets of sampling equipment of the same design of those used on cows, which were evenly distributed along the stalls at about 1.5 m above the floor and mid-way between adjacent cows. Once removed, canisters had their remaining pressure determined and were then over-pressurized with gaseous nitrogen to 121,423 ± 404 Pa. Gases inside the canisters were allowed to mix for at least 1 h prior to transferring four subsamples to pre-evacuated vials for gas composition determination.

Methane measurements were recorded every 24 h for 6 d during the entire sampling and measurement subperiods. Background concentrations were averaged daily to give a single estimate for each period. Ratios of background gas concentrations to gas concentrations in the samples were <10%.

### 2.6 Rumen sampling

On days 5 and 6 of the measurement and sampling subperiods, 200-mL samples of rumen contents were taken approximately 30 min before feeding, and at 1, 3, 6, 12 and 18 h post-feeding. Rumen contents were strained to separate solids and fluid, and rumen pH (ExStik® pH Meter, Extech Instruments, Boston, U.S.) and reducing potential (E*h*, Oakton® pH 700 m, Singapore, equipped with a Schott Instruments BlueLine 31 Rx Ag/AgCl ORP electrode) were immediately measured in the fluid fraction. Triplicate 1-mL aliquots were preserved with 0.2 mL 20% (*m*/*v*) meta-phosphoric acid for determination of volatile fatty acids (VFA) concentration and with 0.2 mL 1% (*v*/*v*) sulfuric acid for subsequent analysis of NH_4_^+^ concentration. Rumen fluid samples were kept frozen at -20 °C until analyzed.

### 2.7 In situ incubations

On day 2 of each measurement and sampling subperiod, and immediately after the morning feeding, seven nylon bags for in situ incubation were placed in the rumen of each animal: three bags, each containing 10 g DM of cotton lint (94.9% DM, and 99.9% organic matter (OM), 0.5% CP and 97% neutral detergent fiber (NDF), on a DM basis), three bags each containing 30 g DM ryegrass hay previously ground through a 2 mm sieve (88.4% DM, and 94.0% OM, 6.3% CP and 68.0% NDF, on a DM basis), and one bag left empty as a blank. The exact weight of each bag and substrate were recorded. The seven bags were attached to the inner side of the cannula stopper by a 60-cm cord. One bag with each substrate remained in the rumen for 12, 24 or 48 h of incubation, whereas the blank was removed at 48 h of incubation. Once removed, bags were gently rinsed with tap water and frozen at -20 °C until processed.

### 2.8 Analyses

Samples of silage, concentrate mixture, refusals and feces were thoroughly homogenized, oven-dried at 60 °C for 48 h, ground through a 1 mm sieve, and analyzed for DM, total ash and CP (AOAC, 2005). Neutral detergent fiber content was assayed with a heat stable amylase and expressed inclusive of residual ash (Mertens, 2002). Acid detergent fiber (ADF) content was determined and expressed inclusive of residual ash (AOAC, 2005). Gross energy of silage, concentrate mixture, refusals, feces, urine and milk, was determined by oxygen bomb calorimetry (Bateman, 1970).

In situ bags were thawed and thoroughly washed under cold tap water until the water running off was clear, after which they were freeze-dried, weighed, and the content of the bags was homogenized. Residues of incubated hay and cotton balls were analyzed for DM content (AOAC, 2005). Residues of incubated ryegrass hay were also analyzed for NDF content (Mertens, 2002). Cotton balls 48 h incubation residues were analyzed for total N (AOAC, 2005), individual AA (except for Tyr, Trp, Cys, Asn and Gln) (White et al., 1986), and long chain fatty acids (AOAC, 2005).

Gas samples were analyzed for CH_4_ and SF_6_ concentration by using a GC (Perkin Elmer Clarus 600, Perkin Elmer, Waltham, USA). Methane was analyzed with a Carboxen 1010 plot column (15 m × 0.32 mm ID, Supelco, Sigma-Aldrich, St. Louis, U.S.) and a flame ionization detector operating at 250 °C. Sulfur hexafluoride was analyzed with an Elite-GC GS Molesieve column (30 m × 0.53 mm ID × 50-μm film thickness, PerkinElmer, Waltham, U.S.) and an electron capture detector operated at 300 °C. Ratio of SF_6_ to CH_4_ in gas samples was equal to 2.04 ± 1.12 (mean ± SD) ppt/ppm. Volatile fatty acids concentration was determined in a GC (Perkin Elmer Clarus 580, Perkin Elmer, Waltham, US) equipped with an Elite-FFAP (PerkinElmer, Shelton, CT, USA) capillary column and a flame ionization detector (Ungerfeld et al., 2019). Rumen NH_4_^+^ concentration was determined according to Kaplan (1969). Urine samples were analyzed for purine derivatives (allantoin and uric acid) by HPLC (Vlassa et al., 2009) and total N (AOAC, 2005). Milk samples were analyzed for fat, protein, lactose, urea and somatic cell counts using infrared spectroscopy (IS, MilkoScan 4000, Foss Electric, Hillerød, Denmark).

### 2.9 Calculations

Composition of the diet ingested was calculated by subtracting the amount of each dietary fraction refused from its amount offered. Ingestion of digestible energy (DE) was calculated as ingestion of GE minus GE output in feces. Ingestion of metabolizable energy (ME) was calculated as ingestion of DE minus GE losses in CH_4_ and urine (McDonald et al., 2010). Feed content of DE and ME was calculated as ingestion of DE and ME, respectively, divided by DM intake (DMI). Energy-corrected milk production (ECM) was calculated according to Tyrrell and Reid (1965). Calculation of CH_4_ production per animal considered background concentrations of CH_4_ and SF_6_ (Muñoz et al., 2019). Methane production was averaged to obtain one average value per cow and period for the statistical analysis. Methane yield was calculated as the quotient between CH_4_ production and DMI. The cotton balls substrate incubated in situ was devoid of N, AA and long chain fatty acids, therefore, N, AA and long chain fatty acids present in the 48 h incubation residues are assumed to be entirely microbial. Microbial N production was calculated from purine derivatives excretion in urine according to IAEA (1997). The E*h* values recorded for rumen fluid were corrected to the Standard Hydrogen Electrode (SHE) by adding 197 mV (Sawyer et al., 1995).

### 2.10 Statistical analyses

Nutrient intake and digestibility, milk production and composition, CH_4_ emissions, energy and N utilization efficiency, and microbial production of N, AA and long chain fatty acids in cotton balls incubated in situ in the rumen, were analyzed with the following linear mixed model:

Response = overall mean + N source + Period + N source × Period + cow (random) + error

With N source being Plant protein or Urea, and Period being Control or 3-NOP administration (with the 3-NOP administration effect being confounded with unintended dietary changes; see 3. Results and 4. Discussion).

When interactions were significant (P < 0.05), treatment means were compared by using Tukey's honestly significant difference contrasts.

In situ digestibility of cotton balls and ryegrass hay was analyzed as a function of:

Response = overall mean + N source + Period + Length of incubation + N source × Period + N source × Length of incubation + Period × Length of incubation + N source × Period × Length of incubation + cow (random) + error

Rumen variables were analyzed with Time after feeding as the repeated measures variable with an asymptotic unbounded variance-covariance structure. Digestible OM intake (DOMI) was included as a covariable to adjust the effects of N source, Period, Time after feeding and their interactions for changes in diet fermentability:

Response = overall mean + N source + Period + Time after feeding + N source × Period + N source × Time after feeding + Period × Time after feeding + N source × Period × Time after feeding + DOMI + cow (random) + Day + cow × Period × Day (random) + error

When interactions with Time after feeding were significant (P < 0.05), treatment means were compared within each time point.

Rumen E*h* was regressed as a function of rumen pH and the experimental treatments, as follows:

E*h* = intercept + N source + Period + N source × Period + pH + pH^2^ + N source × pH + Period × pH + N source × Period × pH + error

Non-significant (P > 0.05) interactions were removed, and the reduced model was re-fitted.

In all statistical analyses, significance was declared at P < 0.05 and tendencies at 0.05 ≤ P < 0.10. All statistical analyses were conducted using JMP® (JMP, 2016).

## 3 Results

### 3.1 Composition of diet offered and ingested

There was high variation among and within silage bales in DM and CP content, especially in the 3-NOP period (Supplementary Figures 1 and 2). Samples from bales which were analyzed prior to the experiment and used to formulate the diets had lower DM content than the actual silage offered in the cows´ feeders in the 3-NOP period. This resulted in greater proportion of silage in the diet DM offered in the 3-NOP period. Lower quality of silages offered in the 3-NOP period resulted in diets offered in the 3-NOP period containing more DM, NDF and ADF than in the Control period (Supplementary Table 1). Crude protein content in the diet ingested was lower in the 3-NOP period with the urea-containing diet. With both N sources and in both periods, CP was considerably lower than the 16% DM basis targeted (Table 2). The same as the diet offered, the diet ingested was also higher in NDF and ADF in the 3-NOP period with both diets, and lower in CP in the 3-NOP period with the Urea diet.

**Table 2:** Proximate composition of diets ingested by dairy cows fed a plant protein only (Plant protein) or a urea-containing (Urea) diet (n = 4), supplemented a placebo (Control period) or the methanogenesis inhibitor 3-nitrooxypropanol (3-NOP period).

N source	Plant Protein		Urea		SEM^1^	N source P =	Period P =	N source × Period P =
Period	Control	3-NOP	Control	3-NOP				
%DM	40.8	43.0	41.5	42.7	1.21	0.88	0.16	0.68
OM (%DM)	95.4	95.7	95.2	95.7	0.095	0.35	0.005	0.23
CP (%DM)	11.9^b^	11.6^b^	13.3^a^	10.4^c^	0.41	0.018	<0.001	<0.001
NDF (%DM)	28.2	41.7	28.3	40.1	0.49	0.023	<0.001	0.069
ADF (%DM)	18.5	23.7	18.3	22.8	0.50	0.27	<0.001	0.54
GE (MJ/kg DM)	19.6^a^	19.0^b^	18.4^c^	18.4^c^	0.043	<0.001	<0.001	<0.001

^1^Standard error of the mean; ^2^Unlike superscripts on the same row indicate significantly (P < 0.05; Tukey HSD) different treatment means when the interaction N source by Period is significant (P < 0.05).

### 3.2 Intake and digestibility

There were no effects of N source on nutrients intake (P ≥ 0.77; Table 3). Dry matter, OM, CP, NDF and GE intake were greater in the Control than in the 3-NOP period (P ≤ 0.015). There was an interaction (P = 0.017) between N source and Period on CP intake, with a 38% lower CP intake in the 3-NOP period than in the Control period with the Urea diet (P < 0.05).

**Table 3:** Intake and apparent digestibility of dry matter and dietary fractions of dairy cows fed a plant protein only (Plant protein) or a urea-containing (Urea) diet (n = 4), supplemented a placebo (Control period) or the methanogenesis inhibitor 3-nitrooxypropanol (3-NOP period).

N source	Plant Protein		Urea		SEM^1^		N source P =	Period P =		N source × Period P =
Period	Control	3-NOP	Control	3-NOP						
Total intake (kg/d or MJ/d)										
DM	17.6	14.9	18.6	14.6	1.58		0.89		0.003	0.39
OM	16.8	14.2	17.7	13.9	1.51		0.89		0.004	0.42
CP	2.09^ab 2^	1.74^ab^	2.46^a^	1.52^b^	0.19		0.77		<0.001	0.017
NDF	4.98	6.21	5.26	5.82	0.53		0.95		0.015	0.26
ADF	3.26	3.55	3.41	3.32	0.35		0.93		0.63	0.37
GE	344	283	342	267	29.9		0.83		0.003	0.66

Digestibility (%)										
DM	67.0	68.3	69.8	70.7	1.00	0.066			0.24	0.87
OM	70.0	70.9	72.3	73.0	0.95	0.090			0.37	0.95
CP	42.4^b^	54.3^a^	50.4^a^	53.8^a^	2.54	0.047			<0.001	0.015
NDF	27.8	54.4	35.0	56.0	3.07	0.26			<0.001	0.30
ADF	15.1	44.9	25.1	48.1	3.70	0.15			<0.001	0.35
GE	68.5	71.3	67.7	75.3	1.39	0.32			0.002	0.063

^1^Standard error of the mean; ^2^Unlike superscripts on the same row indicate significantly (P < 0.05; Tukey HSD) different treatment means when the interaction N source by Period is significant (P < 0.05).

Apparent digestibility of NDF (P < 0.001; Table 3), ADF (P < 0.001) and GE (P = 0.002) were greater in the 3-NOP period. Apparent digestibility of CP was greater in the 3-NOP period with the Plant protein diet (P < 0.05; interaction N source × Period P = 0.015). Intake of apparently digestible DM, OM, NDF, and GE were greater in the Control period (P ≤ 0.024; Supplementary Table 2), and intake of apparently digestible CP was 33% greater in the Control period with the Urea diet (P < 0.05; interaction N source × Period P = 0.004). Intake of digestible ADF was greater in the 3-NOP period (P = 0.003).

### 3.3 Milk production and composition

There were no effects of N source on milk production and composition (P ≥ 0.16; Table 4). Production of milk (P = 0.034), milk fat (P = 0.025), lactose (P = 0.036) and milk GE (P = 0.002) were lower, and production of milk protein tended (P = 0.095) to be lower, in the 3-NOP period compared to the Control period. There were no effects of Period on milk composition, although milk GE content tended to be higher in the Control period (P = 0.069). There were no effects of N source or Period on milk urea N or somatic cell count (P ≥ 0.25).

**Table 4:** Milk production and composition of dairy cows fed a plant protein only (Plant protein) or a urea-containing (Urea) diet (n = 4), supplemented a placebo (Control period) or the methanogenesis inhibitor 3-nitrooxypropanol (3-NOP period).

N source	Plant Protein		Urea		SEM^1^	N source P =	Period P =	N source × Period P =
Period	Control	3-NOP	Control	3-NOP				
Milk (kg/d)	22.0	19.5	22.0	19.5	2.34	0.99	0.034	0.99
Fat (g/kg)	27.8	27.5	30.1	29.9	1.31	0.16	0.81	0.96
Protein (g/kg)	31.9	32.6	33.6	33.4	0.87	0.29	0.67	0.45
Lactose (g/kg)	53.9	52.9	52.7	53.1	0.74	0.60	0.42	0.10
Milk gross energy (MJ/kg)	2.57	2.50	2.73	2.32	0.15	0.98	0.069	0.17
Fat (kg/d)	0.607	0.530	0.663	0.575	0.0634	0.57	0.025	0.85
Protein (kg/d)	0.701	0.637	0.737	0.647	0.0769	0.83	0.095	0.75
Lactose (kg/d)	1.182	1.031	1.160	1.032	0.127	0.96	0.036	0.83
Gross energy in milk (MJ/d)	56.8	46.5	61.5	41.3	6.28	0.98	0.002	0.15
Milk urea nitrogen (mg/dL)	20.5	23.9	26.0	25.3	2.08	0.26	0.25	0.092
Log_10_ SCC^2^	6.42	6.37	6.35	6.27	6.07	0.56	0.43	0.84

^1^Standard error of the mean; ^2^Log-transformed (base 10) somatic cell count.

### 3.4 Methane production

Out of 96 (8 cows × 6 d × 2 periods) CH_4_ measurements, two were eliminated because of capillary tube leakage (as evidenced by atmospheric pressure of the canister at the end of the collection period) and another four measurements were eliminated because their SF_6_/CH_4_ ratios were greater or lesser than the 97.5 or 2.5 percentiles, respectively, of the SF_6_/CH_4_ ratio distribution. Due to this, CH_4_ production results from one cow from the Urea diet were removed because of insufficient days of measurement in the Control period. Results of a second cow from the Plant protein diet were also removed because of supra-physiological CH_4_ production in the Control period (average CH_4_ production of 664 g/d). Methane yield tended (P = 0.067) to be greater, and *Y*_*m*_ was greater (P = 0.002), with the Urea, than with the Plant protein diet. Methane production (−62.1%; P < 0.001; Table 5), CH_4_ yield (−53.5%; P < 0.001), CH_4_ production per kilogram of digested OM (−53.9%; P < 0.001), CH_4_ emissions intensity (−59.1%; P < 0.001), CH_4_ emissions intensity on an ECM basis (−48.7%; P < 0.001), and the proportion of ingested GE lost as CH_4_ (−50.8%; P < 0.001), all decreased in the 3-NOP period.

**Table 5:** Methane emissions of dairy cows fed a plant protein only (Plant protein) or a urea-containing (Urea) diet (n = 4), supplemented a placebo (Control period) or the methanogenesis inhibitor 3-nitrooxypropanol (3-NOP period).

N source	Plant Protein		Urea		SEM^1^	N source P =	Period P =	N source × Period P =
Period	Control	3-NOP	Control	3-NOP				
Measurement days (d/cow) [mean, (range)]	6 (6–6)	5.33 (5–6)	5.67 (5–6)	6 (6–6)	-	-	-	-
CH_4_ (g/d)^2^	458	214	564	190	66.4	0.65	<0.001	0.13
CH_4_ (g/d)^3^	389	160	476	167	30.5	0.28	<0.001	0.14
CH_4_ yield (g/kg DMI)^3^	21.5	10.1	23.9	11.0	0.56	0.067	<0.001	0.18
CH_4_ (g/kg digestible OM intake)^3^	32.6	15.1	34.7	15.9	1.59	0.89	<0.001	0.49
*Y*_*M*_ (MJ CH_4_/100 MJ GE intake)^3^	6.10	2.97	7.20	3.57	0.23	0.002	<0.001	0.13
CH_4_ emissions intensity (g/kg milk)^3^	17.9	7.63	21.4	8.43	0.91	0.12	<0.001	0.13
CH_4_ emissions ECM^4^ intensity (g/kg ECM)^3^	21.6	10.6	23.3	12.4	1.31	0.33	<0.001	0.93

^1^Standard error of the mean; ^2^All animals; ^3^Results with outliers (animals with supra-physiological CH_4_ production) removed from the analysis; ^4^Energy-corrected milk production.

### 3.5 Energy balance

There were no effects of N source on energy balance variables (P ≥ 0.32; Supplementary Table 3). Digestible energy intake was lower in the 3-NOP period (P = 0.024), whereas there was no effect of Period on ME intake (P = 0.25). Nitrogen source and Period interacted on dietary content of DE (P = 0.029) and ME (P = 0.040), which was higher in the 3-NOP period with the Urea diet (P < 0.05).

### 3.6 Nitrogen balance

Both in the Control and 3-NOP periods, the Plant protein and the Urea diets resulted in negative N balance (Table 6); all individual animals were in negative N balance in both periods as well (result not shown). Nitrogen output in feces (P < 0.001) and manure (P = 0.002) was greater in the Control period, whilst there were no effects of N source (P = 0.50) or Period (P = 0.21) on N output in urine or on retained N (P ≥ 0.53). More N was eliminated in urine as a proportion of N intake in the 3-NOP period (P = 0.013). There was no effects of N source or Period on microbial N production estimated through purine derivatives excretion (P ≥ 0.35). The proportion of ingested N secreted in milk was higher in the 3-NOP period (P < 0.05) with the Urea diet (interaction P = 0.047).

**Table 6:** Nitrogen balance of dairy cows fed a plant protein only (Plant protein) or a urea-containing (Urea) diet (n = 4), supplemented a placebo (Control period) or the methanogenesis inhibitor 3-nitrooxypropanol (3-NOP period).

N source	Plant Protein		Urea		SEM^1^	N source P =	Period P =	N source × Period P =
Period	Control	3-NOP	Control	3-NOP				
N ingested (g/d)	334^ab2^	278^ab^	394^a^	247^b^	31.0	0.77	<0.001	0.017
N in feces (g/d)	194	128	197	113	19.2	0.83	<0.001	0.36
N in urine (g/d)	114	115	135	113	10.8	0.50	0.21	0.18
N in manure^3^ (g/d)	307	242	332	226	28.6	0.92	0.002	0.25
N in milk (g/d)	112	94.7	118	94.1	11.7	0.85	0.014	0.57
Retained N (g/d)	-84.8	-59.0	-55.9	-72.5	11.1	0.53	0.68	0.094
N in feces/N ingested (g/g)	0.576^a^	0.457^b^	0.496^b^	0.454^b^	0.0138	0.032	<0.001	0.020
N in urine/N ingested (g/g)	0.345	0.427	0.342	0.461	0.0321	0.68	0.013	0.55
N in feces/N in urine (g/g)	1.69	1.13	1.46	1.00	0.099	0.21	<0.001	0.36
N in manure^3^/N ingested (g/g)	0.921	0.885	0.837	0.915	0.0353	0.43	0.61	0.19
N in milk/N ingested (g/g)	0.331^ab^	0.335^ab^	0.302^b^	0.387^a^	0.0211	0.66	0.034	0.047
Microbial N production^4^ (g/d)	153	136	145	141	19.7	0.95	0.35	0.52

^1^Standard Error of the Mean; ^2^Unlike superscripts on the same row indicate significantly (P < 0.05; Tukey HSD) different treatment means when the interaction N source by Period is significant (P < 0.05); ^3^Feces + urine; ^4^Estimated from the excretion of purine derivatives (IAEA, 1997).

### 3.7 In situ incubations

Apparent in situ DM disappearance from cotton balls at 12, 24 and 48 h incubation was greater in the 3-NOP period with both diets (P < 0.001; Supplementary Figure 3). There was no effect of the N source (P = 0.79) or Period (P = 0.13) on DM disappearance of ryegrass hay (Supplementary Figure 4). There was greater NDF disappearance of ryegrass hay in the 3-NOP period (P = 0.018), with no effect of N source (P = 0.78; Supplementary Figure 5).

In situ microbial N and total microbial AA, Ser, Gly, His, Arg, Tre, Ala, Pro, Val and Met in cotton balls were lower in the 3-NOP period (P ≤ 0.046; Supplementary Table 4). There were no effects of N source or Period on total microbial long chain fatty acids in cotton balls (P ≥ 0.13; Supplementary Table 5). Microbial C18:1 and total monounsaturated long chain fatty acids were greater with the Plant protein diet (P ≤ 0.016). Microbial C14:0 and total polyunsaturated long chain fatty acids were greater in the Control period (P ≤ 0.029).

### 3.8 Rumen variables

**Figure 1:**
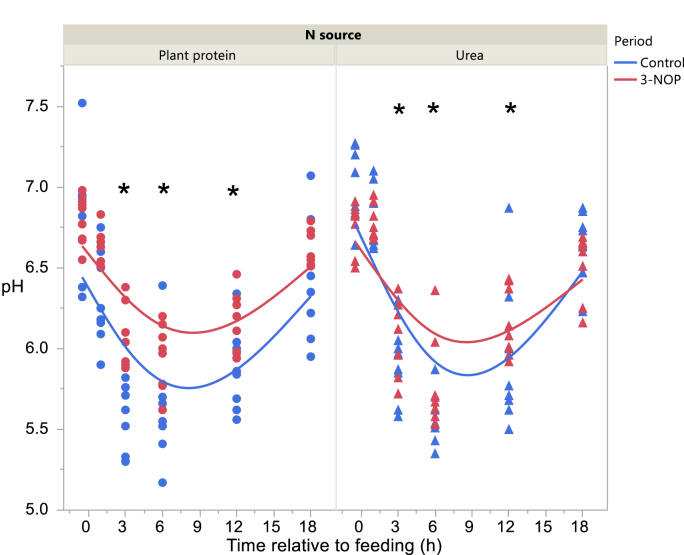
Daily evolution of rumen pH of dairy cows fed a plant protein only or a urea-containing diet, in the Control period or in the 3-NOP supplementation period. Circles correspond to the Plant protein diet and triangles to the Urea diet. Blue symbols and blue curves correspond to the Control period and red symbols and red curves correspond to the 3-NOP period. Significance of fixed effects: N source (N), P = 0.36; Period (Pd), P = 0.040; Time after feeding (T), P < 0.001; N × Pd, P = 0.088; N × T, P < 0.001; Pd × T, P < 0.001; N × Pd × T, P = 0.31; Day, P = 0.032; digestible organic matter intake, P = 0.71. Asterisks (∗) indicate significant differences (P < 0.05) between periods at specific time points.

Rumen pH after feeding was higher in the 3-NOP period (interaction Period × Time after feeding P < 0.001; Figure 1). Rumen E*h* was higher in the 3-NOP period before feeding and 1 h after feeding (P < 0.05; interaction Period × Time after feeding P = 0.002; Supplementary Figure 6). There was a negative quadratic relationship between rumen E*h* and pH (Supplementary Figure 7).

**Figure 2:**
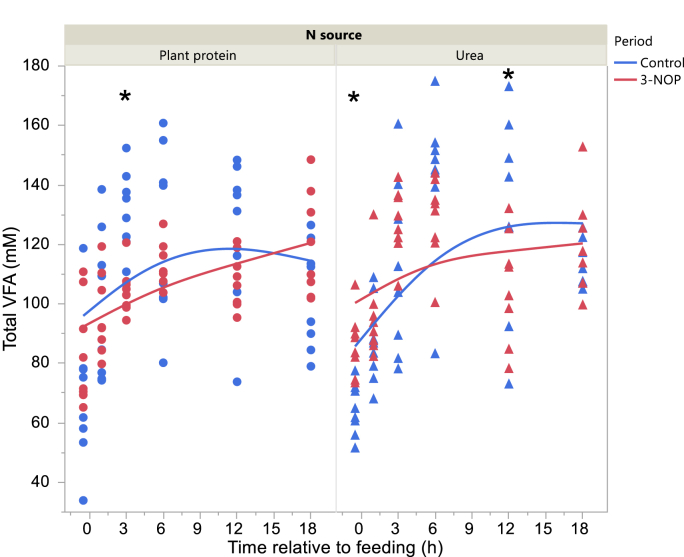
Total VFA concentration in the rumen of dairy cows fed a plant protein only or a urea-containing diet, in the Control period or in the 3-NOP supplementation period. Circles correspond to the Plant protein diet and triangles to the Urea diet. Blue symbols and blue curves correspond to the Control period and red symbols and red curves correspond to the 3-NOP period. Significance of fixed effects: N source (N), P = 0.58; Period (Pd), P = 0.70; Time after feeding (T), P < 0.001; N × Pd, P = 0.44; N × T, P = 0.10; Pd × T, P < 0.001; N × Pd × T, P = 0.010; Day, P = 0.024; digestible organic matter intake, P = 0.72. Asterisks (∗) indicate significant differences (P < 0.05) between periods at specific time points.

**Figure 3:**
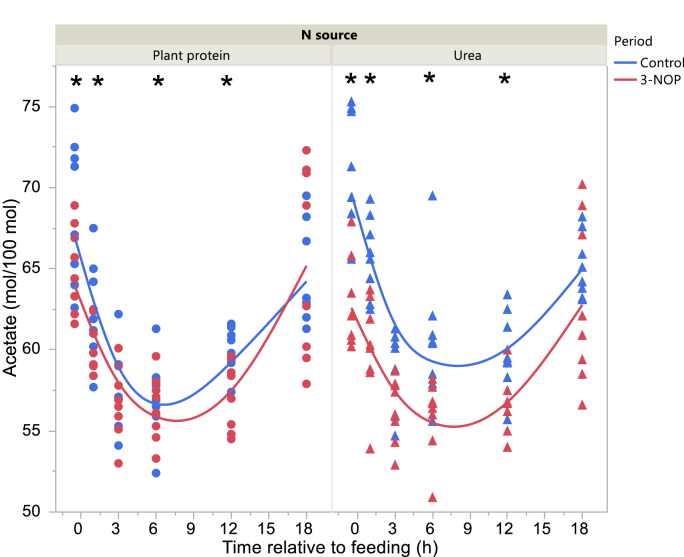
Daily evolution of acetate molar percentage in the rumen of dairy cows fed a plant protein only or a urea-containing diet, in the Control period or in the 3-NOP supplementation period. Circles correspond to the Plant protein diet and triangles to the Urea diet. Blue symbols and blue curves correspond to the Control period and red symbols and red curves correspond to the 3-NOP period. Significance of fixed effects: N source (N), P = 0.60; Period (Pd), P = 0.001; Time after feeding (T), P < 0.001; N × Pd, P = 0.009; N × T, P = 0.61; Pd × T, P = 0.012; N × Pd × T, P = 0.85; Day, P = 0.64; digestible organic matter intake, P = 0.32. Asterisks (∗) indicate significant differences (P < 0.05) between periods at specific time points.

**Figure 4:**
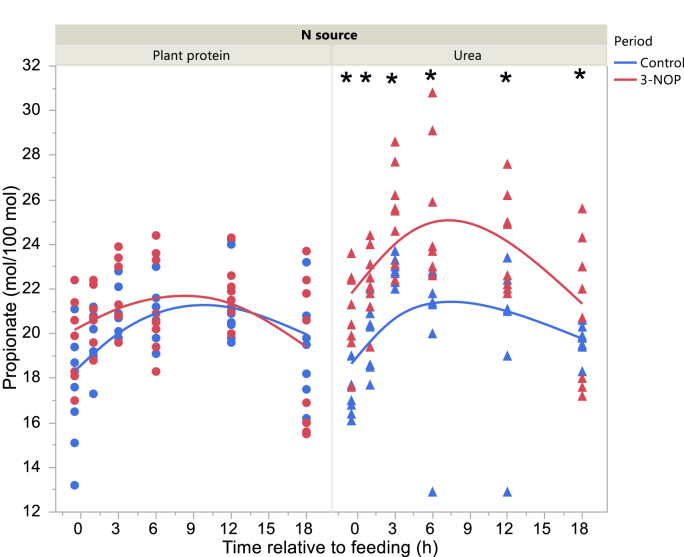
Daily evolution of propionate molar percentage in the rumen of dairy cows fed a plant protein only or a urea-containing diet, in the Control period or in the 3-NOP supplementation period. Circles correspond to the Plant protein diet and triangles to the Urea diet. Blue symbols and blue curves correspond to the Control period and red symbols and red curves correspond to the 3-NOP period. Significance of fixed effects: N source (N), P = 0.071; Period (Pd), P = 0.034; Time after feeding (T), P < 0.001; N × Pd, P = 0.025; N × T, P = 0.080; Pd × T, P = 0.076; N × Pd × T, P = 0.63; Day, P = 0.79; digestible organic matter intake, P = 0.16. Asterisks (∗) indicate significant differences (P < 0.05) between periods at specific time points.

**Figure 5:**
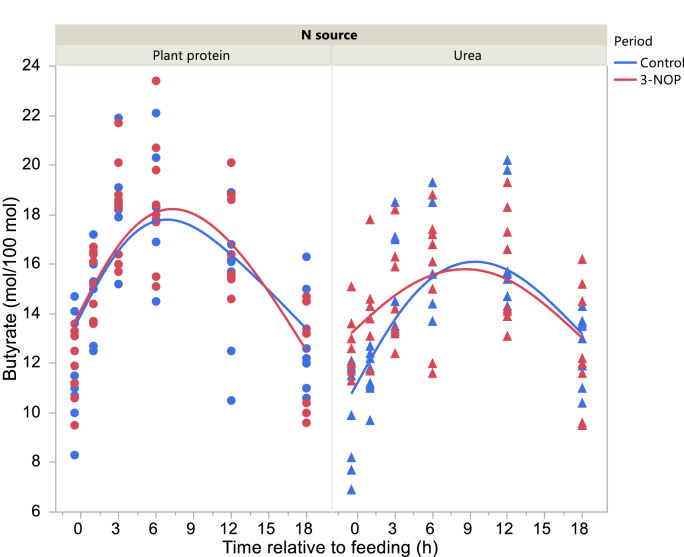
Daily evolution of butyrate molar percentage in the rumen of dairy cows fed a plant protein only or a urea-containing diet, in the Control period or in the 3-NOP supplementation period. Circles correspond to the Plant protein diet and triangles to the Urea diet. Blue symbols and blue curves correspond to the Control period and red symbols and red curves correspond to the 3-NOP period. Significance of fixed effects: N source (N), P = 0.25; Period (Pd), P = 0.005; Time after feeding (T), P < 0.001; N × Pd, P = 0.083; N × T, P < 0.001; Pd × T, P = 0.080; N × Pd × T, P = 0.22; Day, P = 0.82; digestible organic matter intake, P = 0.009.

**Figure 6:**
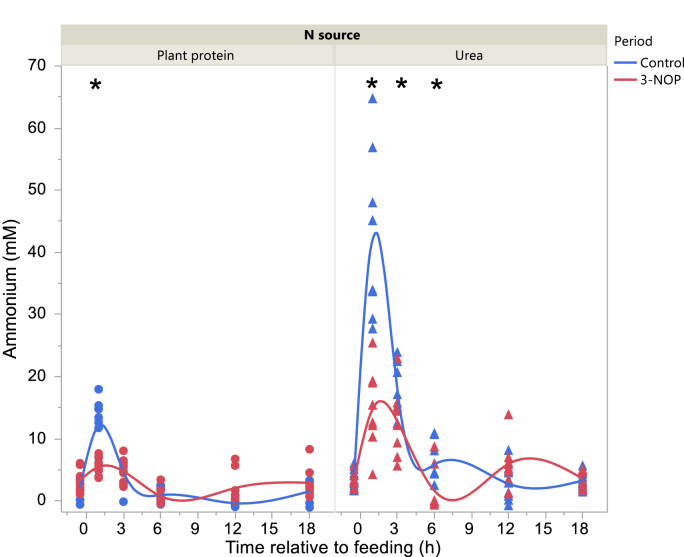
Daily evolution of ammonium concentration in the rumen of dairy cows fed a plant protein only or a urea-containing diet, in the Control period or in the 3-NOP supplementation period. Circles correspond to the Plant protein diet and triangles to the Urea diet. Blue symbols and blue curves correspond to the Control period and red symbols and red curves correspond to the 3-NOP period. Significance of fixed effects: N source (N), P < 0.001; Period (Pd), P = 0.004; Time after feeding (T), P < 0.001; N × Pd, P = 0.003; N × T, P < 0.001; Pd × T, P < 0.001; N × Pd × T, P < 0.001; Day, P = 0.54; digestible organic matter intake, P = 0.79. Asterisks (∗) indicate significant differences (P < 0.05) between periods at specific time points.

Total VFA concentration in the Control period was higher 3 and at 12 h after feeding with the Plant protein diet and Urea diets, respectively, and before feeding with the Urea diet in the 3-NOP period (interaction N source × Period × Time P = 0.010; Figure 2). Acetate molar percentage was lower in the 3-NOP period before feeding and at 1, 6 and 12 h after feeding (interaction Period × Time after feeding P = 0.012; Figure 3). Propionate molar percentage was greater in the 3-NOP period with the Urea diet (P < 0.001) with no differences with the Plant protein diet (P = 0.19; interaction N source × Period P = 0.025; Figure 4). Butyrate molar percentage was greater in the 3-NOP period (P = 0.005; Figure 5). Molar percentages of branched-chain volatile fatty acids were greater in the 3-NOP period at various time points (P < 0.05; interaction Period × Time after feeding P ≤ 0.004; Supplementary Figures 8 and 9). There were no effects of Period on valerate molar percentage (Supplementary Figure 10). Caproate molar percentage was greater in the Control period at 1, 3, 6 and 12 h after feeding (P < 0.05; interaction Period × Time after feeding P < 0.001; Supplementary Figure 11). The acetate to propionate molar ratio was lower in the 3-NOP period (P = 0.005; Supplementary Figure 12). Rumen concentration of NH_4_^+^ was lower in the 3-NOP period 1 h after feeding with the Plant protein diet (P < 0.05), and at 1, 3 and 6 h after feeding with the Urea diet (P < 0.05; interaction N source × Period × Time after feeding P < 0.001; Figure 6).

## 4 Discussion

The high variation among silage bales used in the study resulted in the diets offered in the 3-NOP period being of lower quality compared to the Control period. Therefore, the effect of 3-NOP supplementation is confounded with dietary changes. This problem imposes a limitation at interpreting 3-NOP effects, and we thus opted to refer to “3-NOP period” rather than “3-NOP treatment” in this study. Moreover, diets were N-deficient with both N sources and in both periods. Low CP content resulted in negative N balance in all animals in both periods, which makes difficult evaluating the effects of 3-NOP on N metabolism in this study.

A meta-analysis of five experiments in which 3-NOP was supplemented to dairy cattle reported a mean decrease of 29.6% daily CH_4_ production with the average dose of 81 mg/kg DM (Dijkstra et al., 2018). The greater effect of 3-NOP on CH_4_ production in our study might have been partly related to the somewhat greater dose of 100 mg/kg DM we used, although the higher content of NDF of our diets in the 3-NOP period compared to the average of the diets in the meta-analysis by Dijkstra et al. (2018) would have played in the opposite direction, according to the findings of those authors. Interestingly, all diets in the meta-analysis by Dijkstra et al. (2018) were sufficient in N, with a CP content ranging between 16.1 and 19.6% DM basis; it is possible that in our study the inhibition of methanogenesis by 3-NOP was amplified by N deficiency, an aspect that would have to be confirmed by future studies.

The SF_6_ technique in ruminally-cannulated animals can induce biases in the estimation of CH_4_ production due to gas leaking through the rumen cannula (Beauchemin et al., 2012), however, the decrease in CH_4_ in the 3-NOP period was considerably greater than the biases in CH_4_ production reported by those authors, and cows remained with the same cannula and under the same management scheme throughout the experiment, as their own controls. It is therefore not thought that our conclusions with regards to the effects of 3-NOP on the extent of methanogenesis inhibition were influenced by the use of cannulated animals.

**Figure 7:**
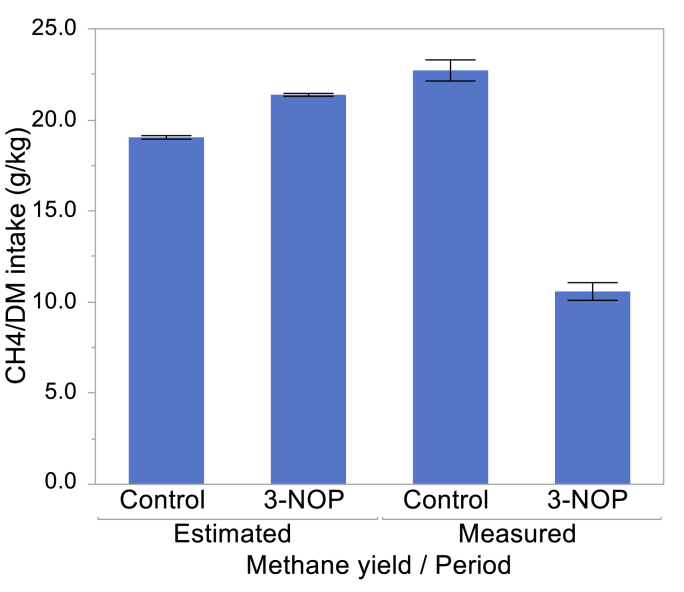
Methane yield estimated from dietary NDF content according to Niu et al. (2018), and CH_4_ yield actually measured, in the Control and 3-NOP periods.

Roughages result in greater CH_4_ yield and *Y*_*m*_ compared to concentrates (Johnson and Johnson, 1995; Beauchemin et al., 2020). Based on the model by Niu et al. (2018), the increase in dietary NDF content that occurred in the 3-NOP period would have been expected to increase CH_4_ yield by 12%; instead, CH_4_ yield decreased by 54% (Figure 7), demonstrating that the observed effects on methanogenesis were due to 3-NOP supplementation rather than to the unintended changes in diet composition. Also, roughages are fermented in the rumen to higher acetate to propionate molar ratio compared to concentrates (Janssen, 2010). In the present study, however, even though the diet contained higher NDF in the 3-NOP period, the rumen fermentation profile was lower in acetate and higher in propionate in the 3-NOP than in the Control period. A decrease in the rumen acetate to propionate ratio as a consequence of 3-NOP administration has been observed in several studies (Jayanegara et al, 2018). It is of much interest that supplementation with 3-NOP could overcome the greater CH_4_ production potential and rumen fermentation characteristics of the lower quality diet offered in the 3-NOP period. It is also of much interest that the decrease of CH_4_ emissions exerted by 3-NOP was similar with a plant protein-based and a urea-supplemented diet.

We hypothesized that part of the metabolic hydrogen spared from methanogenesis would be redirected towards the incorporation of NH_4_^+^ into the synthesis of microbial AA. In agreement with our hypothesis, the post-feeding NH_4_^+^ peak was substantially lower in the 3-NOP period irrespectively of the N source. With the Urea diet, considerably greater ingestion of total and digestible CP in the Control period contributes to explain the lower NH_4_^+^ peak after feeding in the 3-NOP period; however, with the Plant protein diet, ingestion of digestible CP was similar in both periods, and yet the 1 h post-feeding NH_4_^+^ concentration was 55% lower in the 3-NOP period also with the Plant protein diet.

Lower rumen NH_4_^+^ concentration as a result of 3-NOP supplementation agrees with the meta-analyses by Jayanegara et al. (2018) and Kim et al. (2020). However, the moderation observed in the post-feeding NH_4_^+^ peak in the 3-NOP period contrasts with the lack of effects of 3-NOP supplementation on microbial N production, the increase in the proportion of ingested N excreted in urine in the 3-NOP period, and the lower total microbial N and AA in cotton balls incubated in situ in the 3-NOP period. Also, increased molar percentage (and also concentration; results not shown) of branched-chain VFA, which are fermentation products of branched chain AA (Allison, 1978), in the 3-NOP period, does not suggest an increase in the amination of branched-chain VFA, or an inhibition in the deamination of branched-chain AA, in the 3-NOP period.

Lower fermentability of the diets fed in the 3-NOP period may have limited microbial protein synthesis (Firkins et al., 2007; Hartinger et al., 2018; Firkins and Mackie, 2020), in agreement with the lack of response in AA synthesis to methanogenesis inhibition observed in rumen mixed cultures growing in cellulose, in contrast to those growing on starch (Ungerfeld et al., 2020). Furthermore, all diets were N-limited, as evidenced by the overall negative N balance. Hence, a response in the incorporation of NH_4_^+^ into AA synthesis and thus microbial protein production, could have also been limited by rumen NH_4_^+^ concentration, which was below the minimal range of 5 to 11 mM (Schwab and Broderick, 2017) during much of the day in all periods and diets. Unfortunately, the unintended differences between diets in energy and N, and the deficiencies in dietary N and rumen NH_4_^+^, do not allow to conclude on whether inhibiting methanogenesis with 3-NOP might have stimulated the incorporation of NH_4_^+^ into microbial AA synthesis. Whilst the observed effects of 3-NOP on rumen NH_4_^+^ post-feeding concentration are of much interest, they need to be confirmed with further experiments with balanced diets covering animal and microbial requirements of energy and N.

Another aspect to consider when interpreting the lack of response to 3-NOP of microbial protein production, and the lower production of microbial N and AA in cotton balls incubated in situ in the 3-NOP period, is the unknown adaptation time needed to evaluate the hypothesized effects of inhibiting methanogenesis on rumen N metabolism. While the shortened 10-d adaptation period to 3-NOP was sufficient for observing a strong decrease in CH_4_ production, it is possible that changes in non-archaeal microbial populations with noticeable influence on amination and deamination could take longer to occur, and may require a longer adaptation period to be detected.

There has been speculation that an increase in microbial long chain fatty acids synthesis can contribute to explain for unaccounted reducing equivalents when methanogenesis is inhibited in rumen fermentation (Ungerfeld, 2015). In the present study, a response in long chain fatty acids in cellulose incubated in situ was not confirmed, although direct incorporation of long chain fatty acids by microbes colonizing cotton balls cannot be discarded. Whether this result may change balancing the diets offered or with different in situ incubated substrates remains to be investigated.

The differences between periods in milk production cannot be ascribed to 3-NOP supplementation considering the diet composition differences in energy, and the overall N deficiency in both periods and with both N sources. Higher dietary fiber content in the 3-NOP period resulted in lower feed intake likely because of lower rumen outflow rates (Allen, 2014). Likewise, greater fiber digestibility in the 3-NOP period was likely the result of lower feed intake increasing rumen retention times (Illius and Allen, 1994).

All combinations of N sources and periods resulted in milk fat depression. Milk fat depression can be caused by excess dietary fermentable carbohydrates and low physically effective fiber, or by excess dietary supplementation of unsaturated fatty acids (Harvatine et al., 2009; Dewanckele et al., 2020). Because diets were not supplemented with fats or oils, and the rapeseed meal supplement used was solvent-extracted, it seems likely that in this study milk fat depression resulted from the relatively high dietary content of concentrates, which constituted about half of the dietary DM.

Previous in vitro rumen fermentation studies had reported decreases in E*h* as a consequence of the inhibition of methanogenesis, or lack of effects in some studies and treatments (Sauer and Teather, 1987; Soliva et al., 2011; Ungerfeld et al., 2019, 2020). Therefore, higher E*h* in the 3-NOP period before and right after feeding was unexpected. The present results also contrast with the meta-analysis by Huang et al. (2018), in which diets lower in concentrates and higher in NDF were associated with lower E*h*, although they concur with the negative relationship between pH and E*h* reported by Huang et al. (2018).

## 5 Conclusions

Unintended changes in diet composition between the Control and 3-NOP periods, as well as severe limitations in rumen NH_4_^+^ concentration and negative N balance in all animals in both experimental periods preclude us from unequivocally concluding about the effects of inhibiting methanogenesis with 3-NOP on the incorporation of NH_4_^+^ into microbial AA and protein synthesis. Observations of the effects of 3-NOP on rumen ammonium are of interest and deserve further study with isoenergetic and isonitrogenous diets matching animal and microorganisms nutrient requirements. Under N limitation, supplementation with 3-NOP was very effective at decreasing CH_4_ emissions with both a plant protein diet and a diet containing urea, with diets higher in fiber and presumably more methanogenic than the diets fed in the Control period.

## Declarations

### Author contribution statement

Florencia Garcia: Performed the experiments; Analyzed and interpreted the data; Wrote the paper.

Camila Muñoz: Performed the experiments; Analyzed and interpreted the data; Contributed reagents, materials, analysis tools or data.

Jorge Martínez-Ferrer, Natalie L. Urrutia, Emilio D. Martínez, Marcelo Saldivia: Performed the experiments.

Irmgard Immig: Conceived and designed the experiments.

Maik Kindermann, Nicola Walker: Contributed reagents, materials, analysis tools or data.

Emilio M. Ungerfeld: Conceived and designed the experiments; Performed the experiments; Analyzed and interpreted the data; Contributed reagents, materials, analysis tools or data; Wrote the paper.

### Funding statement

This work was supported by Agencia Nacional de Investigación y Desarrollo (ANID) projects FONDECYT 1160764, 1190574 and 1191476. F.G. was supported by the CGIAR Research Program on Climate Change, Agriculture and Food Security (CCAFS) and the Global Research Alliance on Agricultural Greenhouse Gases (GRA) through their CLIFF-GRADS program.

### Data availability statement

The authors do not have permission to share data.

### Declaration of interest’s statement

The authors declare the following conflict of interests

Emilio M. Ungerfeld reports a relationship with DSM Nutritional Products AG that includes: consulting or advisory.

Irmgard Immig reports a relationship with DSM Nutritional Products AG that includes: former employment.

Maik Kindermann reports a relationship with DSM Nutritional Products AG that includes: employment.

Nicola Walker reports a relationship with DSM Nutritional Products AG that includes: employment.

Maik Kindermann has patent #WO 2012/084629 Al issued to Stephane Duval and Maik Kindermann.

### Additional information

No additional information is available for this paper.

## Appendix A Supplementary data

Supplemental information clean.docx Supplemental information clean.docx

## Footnotes

2 Abbreviations: 3-NOP = 3-nitrooxypropanol; AA = amino acids; ADF = acid detergent fiber; CH_4_ = methane; CP = crude protein; DE = digestible energy; DM = dry matter; DMI = dry matter intake; ECM = energy-corrected milk; E*h* = reducing potential; E.P.A. = Environmental Protection Agency; GE = gross energy; ME = metabolizable energy; N = nitrogen; NAD^+^ = oxidized nicotinamide adenine dinucleotide; NADH = reduced nicotinamide adenine dinucleotide; NADP^+^ = oxidized nicotinamide adenine dinucleotide phosphate; NADPH = reduced nicotinamide adenine dinucleotide phosphate; NDF = neutral detergent fiber; NH_4_^+^ = ammonium; OM = organic matter; SF_6_ = sulfur hexafluoride; VFA = volatile fatty acids; *Y*_*m*_ = proportion of dietary gross energy lost as methane.
