# Supplementary material for: 3-Nitrooxypropanol substantially decreased enteric methane emissions of dairy cows fed true protein- or urea-containing diets
Source: Heliyon. 2022 Jun 16;8(6):e09738. doi: 10.1016/j.heliyon.2022.e09738 (PMC9234604; doi:10.1016/j.heliyon.2022.e09738)
Supplement: Supplemental information clean.docx [file mmc1.docx]

**3-Nitrooxypropanol substantially decreased enteric methane emissions of dairy cows fed true protein- or urea-containing diets**

Florencia García ^a, †^, Camila Muñoz ^b, †^, Jorge Martínez-Ferrer ^c, †^, Natalie L. Urrutia ^b, †^, Emilio D. Martínez ^d, †^, Marcelo Saldivia ^d, †^, Irmgard Immig ^e, †^, Maik Kindermann ^e, †^, Nicola Walker ^e, †^, Emilio M. Ungerfeld ^f, *^

^a^ Universidad Nacional de Córdoba, Facultad de Ciencias Agropecuarias, Departamento de Producción Animal, Ing Agr. Félix Aldo Marrone 746, Córdoba Capital, Córdoba, 5001, Argentina.

^b^ Instituto de Investigaciones Agropecuarias, Centro Regional de Investigación Remehue, Ruta 5 km 8 norte, Osorno, Los Lagos, 5290000, Chile.

^c^ Instituto Nacional de Tecnología Agropecuaria, Estación Experimental Agropecuaria Manfredi, Ruta Nacional nº9 km 636, Manfredi, Córdoba, 5988, Argentina.

^d^ Facultad de Ciencias Veterinarias, Universidad Austral de Chile, Campus Isla Teja S/N, Valdivia, 5090000, Chile.

^e^ DSM Nutritional Products, Animal Nutrition and Health, Wurmisweg 576, Kaiseraugst, 4303, Switzerland.

^f^ Instituto de Investigaciones Agropecuarias, Centro Regional de Investigación Carillanca, Camino Cajón - Vilcún km 10, Temuco, La Araucanía, 4880000, Chile.

### ^†^ *E-mail addresses*: Florencia García, [fgarcia@agro.unc.edu.ar](mailto:fgarcia@agro.unc.edu.ar); Camila Muñoz, [camila.munoz@inia.cl](mailto:camila.munoz@inia.cl); Jorge Martínez-Ferrer, [martinez.ferrer@inta.gob.ar](mailto:martinez.ferrer@inta.gob.ar); Natalie L. Urrutia, [natalie.urrutia@inia.cl](mailto:natalie.urrutia@inia.cl); Emilio D. Martínez, [emiliomartinez@uach.cl](mailto:emiliomartinez@uach.cl); Marcelo Saldivia, [marcelosaldiviamv@gmail.com](mailto:marcelosaldiviamv@gmail.com); Irmgard Immig, [irmgard.immig@gmail.com](mailto:irmgard.immig@gmail.com); Maik Kindermann, [maik.kindermann@dsm.com](mailto:maik.kindermann@dsm.com); Nicola Walker, nicola.walker@dsm.com

^*^ Corresponding author. *E-mail address*: [emilio.ungerfeld@inia.cl](mailto:emilio.ungerfeld@inia.cl) (E.M. Ungerfeld)

Supplementary tables

**Supplementary Table 1.** Proximate composition of diets offered to dairy cows fed a plant protein only (Plant protein) or a urea-containing (Urea) diet (n = 4), supplemented a placebo (Control period) or the methanogenesis inhibitor 3-nitrooxypropanol (3-NOP period).

| N source | Plant Protein | | Urea | | SEM^1^ | N source P = | Period  P = | N source × Period  P = |
| --- | --- | --- | --- | --- | --- | --- | --- | --- |
| Period | Control | 3-NOP | Control | 3-NOP |  |  |  |  |
| Total feed offered (kg DM/d) | 18.8 | 18.4 | 19.5 | 18.0 | 1.57 | 0.91 | 0.30 | 0.53 |
| Silage (%DM) | 45.0 | 54.5 | 46.5 | 55.0 | 0.54 | 0.15 | <0.001 | 0.32 |
| %DM | 43.4 | 53.3 | 43.7 | 53.1 | 0.44 | 0.95 | <0.001 | 0.24 |
| OM (%DM) | 95.0^a 2^ | 95.0^ab^ | 94.8^b^ | 95.1^a^ | 0.039 | 0.17 | 0.13 | 0.017 |
| CP (%DM) | 12.0^b^ | 12.4^b^ | 13.4^a^ | 11.5^c^ | 0.12 | 0.13 | 0.001 | <0.001 |
| NDF (%DM) | 30.3^a^ | 41.8^b^ | 30.3^a^ | 40.1^b^ | 0.41 | 0.15 | <0.001 | 0.028 |
| ADF (%DM) | 20.2 | 25.2 | 19.9 | 24.2 | 0.30 | 0.087 | <0.001 | 0.24 |
| GE (MJ/kg DM) | 19.3^a^ | 18.7^b^ | 18.3^c^ | 18.1^d^ | 0.014 | <0.001 | <0.001 | <0.001 |

^1^Standard error of the mean; ^2^Unlike superscripts on the same row indicate significantly (P < 0.05; Tukey HSD) different treatment means when the interaction N source by Period is significant (P < 0.05).

**Supplementary Table 2.** Intake of digestible dietary fractions of dairy cows fed a plant protein only (Plant protein) or a urea-containing (Urea) diet (n = 4), supplemented a placebo (Control period) or the methanogenesis inhibitor 3-nitrooxypropanol (3-NOP period).

| N source | Plant Protein | | Urea | | SEM^1^ | N source P = | Period  P = | N source × Period  P = |
| --- | --- | --- | --- | --- | --- | --- | --- | --- |
| Period | Control | 3-NOP | Control | 3-NOP |  |  |  |  |
| Digestible intake (kg/d or kJ/d) | | | | | | | | |
| DM | 11.8 | 10.1 | 12.9 | 10.3 | 1.01 | 0.64 | 0.008 | 0.45 |
| OM | 11.8 | 10.0 | 12.8 | 10.2 | 1.00 | 0.68 | 0.007 | 0.44 |
| CP | 0.88^ab^ | 0.94^ab^ | 1.23^a^ | 0.82^b^ | 0.080 | 0.29 | 0.015 | 0.004 |
| NDF | 1.39 | 3.32 | 1.84 | 3.26 | 0.24 | 0.45 | <0.001 | 0.30 |
| ADF | 0.40 | 1.58 | 0.87 | 1.60 | 0.18 | 0.31 | 0.003 | 0.37 |
| GE | 236 | 201 | 231 | 201 | 20.9 | 0.93 | 0.025 | 0.87 |

^1^Standard error of the mean; ^2^Unlike superscripts on the same row indicate significantly (P < 0.05; Tukey HSD) different treatment means when the interaction N source by Period is significant (P < 0.05).

**Supplementary Table 3.** Energy balance of dairy cows fed a plant protein only (Plant protein) or a urea-containing (Urea) diet (n = 4), supplemented a placebo (Control period) or the methanogenesis inhibitor 3-nitrooxypropanol (3-NOP period)^1^.

| N source | Plant Protein | | Urea | | SEM^2^ | N source P = | Period  P = | N source × Period  P = |
| --- | --- | --- | --- | --- | --- | --- | --- | --- |
| Period | Control | 3-NOP | Control | 3-NOP |  |  |  |  |
| Ingested GE (MJ/d) | 344 | 283 | 342 | 267 | 29.9 | 0.83 | 0.003 | 0.66 |
| Energy in feces (MJ/d) | 108 | 81.3 | 111 | 66.8 | 10.5 | 0.68 | <0.001 | 0.15 |
| Energy in CH_4_ (MJ/d) | 25.6 | 11.8 | 31.7 | 10.9 | 3.79 | 0.61 | <0.001 | 0.19 |
| Energy in urine (MJ/d) | 7.68 | 7.11 | 8.51 | 6.79 | 0.97 | 0.85 | 0.092 | 0.35 |
| Energy in milk (MJ/d) | 56.8 | 46.5 | 61.5 | 41.3 | 6.28 | 0.98 | 0.002 | 0.15 |
| Digestible energy intake (MJ/d) | 236 | 201 | 231 | 200 | 20.9 | 0.93 | 0.024 | 0.87 |
| Metabolizable energy intake (MJ/d) | 202 | 182 | 191 | 183 | 21.6 | 0.86 | 0.25 | 0.61 |
| Digestible energy (MJ/kg DM) | 13.4^ab 3^ | 13.5^ab^ | 12.5^b^ | 13.8^a^ | 0.32 | 0.32 | 0.010 | 0.029 |
| Metabolizable energy (MJ/kg DM) | 11.4^ab^ | 12.2^a^ | 10.2^b^ | 12.6^a^ | 0.42 | 0.41 | 0.002 | 0.040 |

^1^For consistency, all energy balance results are calculated excluding the two cows that were left out of the methane production analysis; ^2^Standard error of the mean; ^3^Unlike superscripts on the same row indicate significantly (P < 0.05; Tukey HSD) different treatment means when the interaction N source by Period is significant (P < 0.05).

**Supplementary Table 4**. Production of microbial nitrogen and amino acids in cotton balls incubated in situ for 48 h in the rumen of dairy cows fed a plant protein only (Plant protein) or a urea-containing (Urea) diet (n = 4), supplemented a placebo (Control period) or the methanogenesis inhibitor 3-nitrooxypropanol (3-NOP period).

| N source | Plant Protein | | Urea | | SEM^1^ | N source P = | Period  P = | N source × Period  P = |
| --- | --- | --- | --- | --- | --- | --- | --- | --- |
| Period | Control | 3-NOP | Control | 3-NOP |  |  |  |  |
| Total microbial N (mg) | 222 | 145 | 159 | 124 | 23.1 | 0.094 | 0.033 | 0.37 |
| Total microbial AA (mg) ^2^ | 619 | 380 | 422 | 333 | 68.9 | 0.10 | 0.035 | 0.29 |
| Asp (mg) | 69.1 | 50.0 | 43.8 | 43.6 | 8.39 | 0.084 | 0.27 | 0.28 |
| Glu (mg) | 110 | 69.2 | 71.5 | 58.7 | 12.9 | 0.084 | 0.061 | 0.31 |
| Ser (mg) | 30.5 | 18.6 | 21.7 | 15.1 | 3.25 | 0.084 | 0.015 | 0.42 |
| Gly (mg) | 43.0 | 24.6 | 30.8 | 20.2 | 4.20 | 0.071 | 0.005 | 0.37 |
| His (mg) | 10.7 | 6.25 | 7.41 | 6.37 | 0.99 | 0.13 | 0.016 | 0.11 |
| Arg (mg) | 15.5 | 7.60 | 11.5 | 8.66 | 1.64 | 0.39 | 0.007 | 0.15 |
| Tre (mg) | 28.7 | 17.8 | 20.3 | 14.6 | 3.12 | 0.088 | 0.021 | 0.42 |
| Ala (mg) | 47.0 | 30.2 | 33.3 | 26.3 | 5.00 | 0.11 | 0.034 | 0.35 |
| Pro (mg) | 78.9 | 33.7 | 56.8 | 29.9 | 8.69 | 0.16 | 0.001 | 0.32 |
| Val (mg) | 36.4 | 23.1 | 24.8 | 19.8 | 4.11 | 0.095 | 0.046 | 0.33 |
| Met (mg) | 4.35 | 2.21 | 3.39 | 2.02 | 0.72 | 0.44 | 0.031 | 0.61 |
| Ile (mg) | 33.4 | 21.6 | 22.3 | 18.6 | 4.06 | 0.11 | 0.081 | 0.34 |
| Leu (mg) | 46.0 | 34.3 | 30.0 | 28.6 | 5.55 | 0.075 | 0.26 | 0.38 |
| Phe (mg) | 27.7 | 17.5 | 18.8 | 16.2 | 3.50 | 0.17 | 0.091 | 0.30 |
| Lys (mg) | 38.1 | 22.6 | 24.9 | 23.0 | 5.57 | 0.27 | 0.14 | 0.24 |

^1^Standard error of the mean; ^2^Tyr, Trp, Cys, Gln and Asn are not reported.

**Supplementary Table 5**. Microbial long chain fatty acid in cotton balls incubated in situ for 48 h in the rumen of dairy cows fed a plant protein only (Plant protein) or a urea-containing (Urea) diet (n = 4), supplemented a placebo (Control period) or the methanogenesis inhibitor 3-nitrooxypropanol (3-NOP period).

| N source | Plant Protein | | Urea | | SEM^1^ | N source P = | Period P = | N source × Period  P = |
| --- | --- | --- | --- | --- | --- | --- | --- | --- |
| Period | Control | 3-NOP | Control | 3-NOP |  |  |  |  |
| Total microbial long chain fatty acids (mg) | 563 | 409 | 383 | 375 | 58.3 | 0.17 | 0.13 | 0.16 |
| C14:0 (mg) | 9.42 | 5.40 | 6.56 | 5.38 | 0.78 | 0.092 | 0.0062 | 0.095 |
| C15:0 (mg) | 8.17 | 6.79 | 5.61 | 6.84 | 0.93 | 0.20 | 0.94 | 0.19 |
| C16:0 (mg) | 80.4 | 60.7 | 57.6 | 57.4 | 8.64 | 0.16 | 0.27 | 0.28 |
| C16:1 (mg) | 7.88 | 8.16 | 5.02 | 7.39 | 1.11 | 0.13 | 0.26 | 0.37 |
| C17:0 (mg) | 4.06 | 3.28 | 2.50 | 2.74 | 0.58 | 0.097 | 0.64 | 0.40 |
| C18:0 (mg) | 390 | 286 | 269 | 261 | 43.1 | 0.12 | 0.21 | 0.29 |
| C18:1 (mg) | 27.2 | 17.5 | 15.5 | 14.9 | 2.42 | 0.012 | 0.054 | 0.083 |
| C18:2 (mg) | 11.6 | 7.59 | 8.04 | 6.09 | 1.65 | 0.15 | 0.097 | 0.55 |
| γ-C18:3 (mg) | 7.15 | 5.10 | 4.47 | 4.69 | 0.76 | 0.066 | 0.25 | 0.16 |
| α-C18:3 (mg) | 8.97 | 2.98 | 3.15 | 2.51 | 1.99 | 0.14 | 0.12 | 0.20 |
| 11, 14-C20:2 (mg) | 2.77 | 2.35 | 1.97 | 2.40 | 0.44 | 0.41 | 0.99 | 0.35 |
| γ-C20:3 (mg) | 5.02 | 3.77 | 3.47 | 3.68 | 0.50 | 0.13 | 0.32 | 0.17 |
| Total saturated (mg) | 492 | 362 | 343 | 333 | 53.1 | 0.12 | 0.21 | 0.28 |
| Total monounsaturated (mg) | 35.5 | 26.4 | 19.6 | 23.2 | 3.42 | 0.016 | 0.43 | 0.086 |
| Total polyunsaturated (mg) | 35.5 | 20.6 | 22.0 | 18.3 | 3.76 | 0.057 | 0.029 | 0.16 |
| Total odd-numbered (mg) | 12.2 | 10.1 | 8.11 | 9.57 | 1.47 | 0.14 | 0.82 | 0.24 |
| Average number of carbon atoms/long chain fatty acid (mol/mol) | 17.6 | 17.6 | 17.6 | 17.6 | 0.0092 | 0.10 | 0.25 | 0.69 |

^1^Standard error of the mean.

Supplementary Figures

**Supplementary Figure 1.** Within bale variation in DM content in the Control period and in the 3-NOP supplementation period. Horizontal lines within the boxes represent median sample values. The ends of the boxes represent to first and third quartile. The horizontal lines at the end of the whiskers represent the first quartiles minus 1.5 times the interquartile range and the third quartile plus 1.5 times the interquartile range. Observations falling outside of the whiskers are represented as isolated points.

**Supplementary Figure 2.** Within bale variation in CP content in the Control period and in the 3-NOP supplementation period. Horizontal lines within the boxes represent median sample values. The ends of the boxes represent to first and third quartile. The horizontal lines at the end of the whiskers represent the first quartiles minus 1.5 times the interquartile range and the third quartile plus 1.5 times the interquartile range. Observations falling outside of the whiskers are represented as isolated points.

**Supplementary Figure 3**. Apparent dry matter digestibility (DMD) of cotton balls incubated in situ for 12, 24 or 48 h in the rumen of dairy cows fed a plant protein only or a urea-containing diet, in the Control period or in the 3-NOP supplementation period. Each error bar is constructed using one standard error of the mean. Significance of fixed effects: N source (N), P = 0.20; Period (Pd), P < 0.001; Time point (Tp), P < 0.001; N × Pd, P = 0.54; N × Tp, P = 0.43; Pd × Tp, P = 0.14; N × Pd × Tp, P = 0.65.

**Supplementary Figure 4**. Apparent dry matter digestibility (DMD) of ryegrass hay incubated in situ for 12, 24 or 48 h in the rumen of dairy cows fed a plant protein only or a urea-containing diet, in the Control period or in the 3-NOP supplementation period. Each error bar is constructed using one standard error of the mean. Significance of fixed effects: N source (N), P = 0.79; Period (Pd), P = 0.13; Time point (Tp), P = 0.021; N × Pd, P = 0.28; N × Tp, P = 0.96; Pd × Tp, P = 0.24; N × Pd × Tp, P = 0.48.

**Supplementary Figure 5**. Apparent neutral detergent fiber digestibility (NDFD) of ryegrass hay incubated in situ for 12, 24 or 48 h in the rumen of dairy cows fed a plant protein only or a urea-containing diet, in the Control period or in the 3-NOP supplementation period. Each error bar is constructed using one standard error of the mean. Significance of fixed effects: N source (N), P = 0.78; Period (Pd), P = 0.018; Time point (Tp), P = 0.008; N × Pd, P = 0.14; N × Tp, P = 0.96; Pd × Tp, P = 0.34; N × Pd × Tp, P = 0.60.

**Supplementary Figure 6.** Daily evolution of rumen E*h* of dairy cows fed a plant protein only or a urea-containing diet, in the Control period or in the 3-NOP supplementation period. Circles correspond to the Plant protein diet and triangles to the Urea diet. Blue symbols and blue curves correspond to the Control period and red symbols and red curves correspond to the 3-NOP period. Significance of fixed effects: N source (N), P = 0.31; Period (Pd), P = 0.28; Time after feeding (T), P < 0.001; N × Pd, P = 0.76; N × T, P = 0.072; Pd × T, P = 0.002; N × Pd × T, P = 0.22; Day, P = 0.055; digestible organic matter intake, P = 0.011. Asterisks (*) indicate significant differences (P < 0.05) between periods at specific time points.

**Supplementary Figure 7.** Relationship between rumen E*h* and pH: E*h* = 73.3 (±29.4; P = 0.013) – 2.62 (±2.27; P = 0.25) N source (if Plant protein) – 14.6 (±2.43; P < 0.001) Period (if Control) – 59.6 (±4.62; P < 0.001) pH + 31.8 (±9.63; P = 0.001) (pH – 6.29)^2^ + error; R^2^ = 0.51). Circles correspond to the Plant protein diet and triangles to the Urea diet. Blue symbols and blue curves correspond to the Control period and red symbols and red curves correspond to the 3-NOP period.

**Supplementary Figure 8.** Daily evolution of isobutyrate molar percentage in the rumen of dairy cows fed a plant protein only or a urea-containing diet, in the Control period or in the 3-NOP supplementation period. Circles correspond to the Plant protein diet and triangles to the Urea diet. Blue symbols and blue curves correspond to the Control period and red symbols and red curves correspond to the 3-NOP period. Significance of fixed effects: N source (N), P = 0.29; Period (Pd), P < 0.001; Time after feeding (T), P < 0.001; N × Pd, P = 0.42; N × T, P = 0.65; Pd × T, P = 0.004; N × Pd × T, P = 0.60; Day, P = 0.040; digestible organic matter intake, P = 0.89. Asterisks (*) indicate significant differences (P < 0.05) between periods at specific time points.

**Supplementary Figure 9.** Daily evolution of 2- plus 3-methylbutyrate molar percentage in the rumen of dairy cows fed a plant protein only or a urea-containing diet, in the Control period or in the 3-NOP supplementation period. Circles correspond to the Plant protein diet and triangles to the Urea diet. Blue symbols and blue curves correspond to the Control period and red symbols and red curves correspond to the 3-NOP period. Significance of fixed effects: N source (N), P = 0.25; Period (Pd), P < 0.001; Time after feeding (T), P = 0.001; N × Pd, P = 0.20; N × T, P = 0.43; Pd × T, P < 0.001; N × Pd × T, P = 0.64; Day, P = 0.087; digestible organic matter intake, P = 0.85. Asterisks (*) indicate significant differences (P < 0.05) between periods at specific time points.

**Supplementary Figure 10.** Daily evolution of valerate molar percentage in the rumen of dairy cows fed a plant protein only or a urea-containing diet, in the Control period or in the 3-NOP supplementation period. Circles correspond to the Plant protein diet and triangles to the Urea diet. Blue symbols and blue curves correspond to the Control period and red symbols and red curves correspond to the 3-NOP period. Significance of fixed effects: N source (N), P = 0.10; Period (Pd), P = 0.74; Time after feeding (T), P < 0.001; N × Pd, P = 0.093; N × T, P = 0.023; Pd × T, P = 0.65; N × Pd × T, P = 0.36; Day, P = 0.62; digestible organic matter intake, P = 0.78. Asterisks (*) indicate significant differences (P < 0.05) between periods at specific time points.

**Supplementary Figure 11.** Daily evolution of caproate molar percentage in the rumen of dairy cows fed a plant protein only or a urea-containing diet, in the Control period or in the 3-NOP supplementation period. Circles correspond to the Plant protein diet and triangles to the Urea diet. Blue symbols and blue curves correspond to the Control period and red symbols and red curves correspond to the 3-NOP period. Significance of fixed effects: N source (N), P = 0.56; Period (Pd), P < 0.001; Time after feeding (T), P < 0.001; N × Pd, P = 0.26; N × T, P = 0.92; Pd × T, P < 0.001; N × Pd × T, P = 0.48; Day, P = 0.085; digestible organic matter intake, P = 0.079. Asterisks (*) indicate significant differences (P < 0.05) between periods at specific time points.

**Supplementary Figure 12.** Daily evolution of the acetate to propionate concentration ratio in the rumen of dairy cows fed a plant protein only or a urea-containing diet, in the Control period or in the 3-NOP supplementation period. Circles correspond to the Plant protein diet and triangles to the Urea diet. Blue symbols and blue curves correspond to the Control period and red symbols and red curves correspond to the 3-NOP period. Significance of fixed effects: N source (N), P = 0.20; Period (Pd), P = 0.005; Time after feeding (T), P < 0.001; N × Pd, P = 0.019; N × T, P = 0.77; Pd × T, P = 0.003; N × Pd × T, P = 0.91; Day, P = 0.69; digestible organic matter intake, P = 0.21. Asterisks (*) indicate significant differences (P < 0.05) between periods at specific time points.
